# Screening for developmental delay in urban Rwandan children: a cross sectional study

**DOI:** 10.1186/s12887-023-04332-3

**Published:** 2023-10-20

**Authors:** Victoire Tuyisenge, Febronie Mushimiyimana, Aimable Kanyamuhunga, Jean Paul Rukabyarwema, Archana A. Patel, Cliff O’Callahan

**Affiliations:** 1 College of Medicine and Health sciences, University of Rwanda, Kigali, Rwanda; 2 Pediatrics and Child Health Department, University Teaching Hospital of Kigali, Kigali, Rwanda; 3 Muhima District Hospital, Kigali, Rwanda; 4 Department of Neurology, Harvard Medical School, Boston, USA; 5 Middlesex Health/University of Connecticut, Middletown and Hartford Connecticut, Middletown, USA

**Keywords:** Early childhood development, Developmental screening, Developmental delay, Sub-Saharan Africa

## Abstract

**Background:**

Systematic or targeted screening for developmental delay (DD) is critical to the early identification of developmental disabilities. With limited available information for urban Rwandan children, this study aimed to determine the prevalence of DD and associated risk factors in infants aged 9 to 16 months living in the urban Rwandan city of Kigali.

**Methods:**

A cross-sectional study was conducted in Rwanda from August to November 2019. A convenience sample of 376 Rwandan parents/caregivers and their children attending urban health centers for their routine immunization visits at 9 and 15 months of age was studied. Parents/caregivers completed the official Kinyarwandan version of the Ages and Stages Questionnaire (ASQ-3) and established cutoffs were used to identify DD. Frequency and percentages were used to summarise the data. Logistic regression analysis was used to identify factors associated with DD.

**Results:**

Of the 358 children screened using the ASQ-3, the overall prevalence of DD was 24.6%, with a 27.2% prevalence among 9–10-month old children and 22.4% prevalence among 15–16-month old children. Delays in the combined group among the domains of gross motor, communication, fine motor, personal social, and problem solving were 12.8%, 2.5%, 8.4%, 1.7% and 7.5%, respectively. Gestational age at delivery and district of origin were most highly associated with DD, with preterm children at significantly higher risk of having DD compared to term children (Adjusted Odd Ratio AOR = 8.3; 95% CI = 2.5–27.4) and children from Nyarugenge District at high risk of DD compared to children from Gasabo district (AOR = 2.15; 95% CI = 1.2–3.9).

**Conclusions:**

The prevalence of ASQ-detectable DD among urban Rwandan children between 9 and 16 months of age was 24.6%, with a high correlation to a history of prematurity and district of origin. This study demonstrates the need for thoughtful health planning regarding integrated developmental surveillance for children, particularly those at high risk, to allow for earlier identification and intervention in the urban area of Kigali, Rwanda.

## Introduction

Developmental delay (DD) is defined as any delay in reaching thinking, social, language, or motor development skills compared to peers from the same population [1]. Early childhood is the best time to identify, attempt to prevent adverse events, and intervene in the areas that are responsible for a child’s delay, which has potential negative consequences for the ability to function across the lifespan [2]. Many modifiable risk factors for DD have been identified and they are more prevalent in low and middle-income countries (LMICs) [3]. The World Bank’s 2018 report recognises that optimising the wellbeing of people in the early years of life is an essential component of the social and functional development of countries and it challenges partners to increase investments in early childhood programmes. Despite this endorsement, only one-quarter of the 250 million children under five have access to preschool [4, 5].

It is estimated that more than 200 million children in LMICs do not develop to their full potential [6] and these countries often have weak or absent systems of universal preventive primary care that would identify developmental delay. Meanwhile, the health and education systems in high-income countries (HICs) offer multiple opportunities to prevent, identify and manage early childhood developmental problems [7]. The capacity to duplicate such systems in LMICs is challenging. The World Health Organisation (WHO) highlights the fact that in LMIC regions the lower global health expenditure per person [8] leads to more out-of-pocket payments as the main health financing source. This naturally limits access to care for poor populations. Esmat Nemati et al., in their systematic review on out-of-pocket money payments in LMICs documented a high mean of out-of-pocket money payments of up to 67% as a percentage of households final consumption expenditure [9], in contrast to 1.3% in Turkey and 5.8% in Switzerland [10]. Additionally, there is a tremendous disparity in the healthcare provider-patient ratio, with medical doctors per 10,000 population ratios of 0.23 in LMICs and 84.27 in HICs [11]. This significant disparity in universal comprehensive preventive health care draws attention to the nature and impact of socioeconomic gradients found both within HICs and LMICs, and between nations. Children, particularly those with delays and disadvantaged by lack of access to adequate health and interventional services, perform less well at school, earn less as adults, have higher fertility rates, and are less able to economically provide for their children, leading to intergenerational poverty transmission [12, 13].

Sapna V. Kumar et al. in their systematic review evaluating home based or clinical based interventions on developmental difficulties examined parental practices in nutrition, stimulation, play and communication, documented improved cognition, language and motor development in the intervention group compared to the control group [14]. PK Maulik et al. in their systematic review, identified low-resource, effective, and simple community-based interventions targeting 0–3 years that can be implemented in the LMICs, including reading, play, tactile stimulation, and music [15]. Developmental interventions in LMICs for children identified with delays will have to rely on these lower-cost strategies, much like existing programs to combat iron deficiency and malnutrition, psychosocial stimulation training for home caregivers of high-risk infants, and establishing community rehabilitation programs [16]. The positive impact of such early childhood intervention programs on youth school performance, adult functioning, and economic power has been previously described [17]. Thus, a country like Rwanda, which declares its intention to dramatically improve the lives of its citizens, can find support in the literature to invest in early childhood programs with a high likelihood of economic return [18, 19].

Limited information on DD is available for Rwandan urban children. Two investigations have been conducted in rural Rwandan areas: one studied prior preterm children at 1–3 years of age which showed a prevalence of 67.4% [20]; the second was part of an Early Childhood Development and Family Baseline Evaluation by UNICEF, which demonstrated that children from the poorest families have higher rates of DD than children from wealthier families [21]. Therefore, this present study aimed to determine the prevalence of DD and associated risk factors among infants aged 9 to 16 months living in the urban Rwandan city of Kigali in order to provide a more representative picture of developmental delay. Our results should help to inform policymakers and program planners working in the area of child health.

## Methods

### Study design and period

This is a cross-sectional study of infants coming to the health center for their standard 9th and 15th month vaccinations over a period of 3 months, from August 2019 to November 2019.

### Study setting

Rwanda is a lower-income developing country with a total surface area of 2630 km^2^ and a population of 13,246,394 in August 2022 [22]. The study took place in the most urbanised city of Rwanda, Kigali, the capital city of Rwanda, in its different health centers located in three administrative districts (Nyarugenge, Gasabo and Kicukiro), where one large health center was selected from each district except in Nyarugenge, which has many health centers, and, thus, two large health centers were selected. Data collection took place as children were brought for their routine immunizations: in Nyarugenge district this occurred at Nyiranuma and Muhima health centers, in Gasabo district at Kimironko health center, and in Kicukiro district at Kicukiro health center.

### Study population

All children aged 9–10 months and 15–16 months who were brought to the selected health centers for their routine national-based childhood vaccinations at 9 and 15 months during the study period were included. Children were excluded if their family care provider declined to participate or was not present, or if they fell outside of the specific age categories selected.

### Sample size and sampling procedure

There are limited available data on DD in LMICs with which to compute the sample size. One study using the same measurement tool in Rwanda documented a high prevalence of 67.4% among high-risk prior premature infants aged 1–3 years from a very rural area [20]. We chose to use a prevalence of 37% based on a study from Chile with 8 months old infants since it more likely reflects a similar general population in a LMIC country [23]. Our single population proportion sample size calculation used a prevalence of 37%, a 95% confidence interval, and a 5% margin of error [24] resulting in an initial sample size of 358 which ,estimating a 5% non-response rate, was adjusted to the final sample size of 376.

Since the vast majority of Rwandan families (> 95%) bring their children to government health centers for their vaccinations, we surmised that we could capture a broad representation of families across the economic strata by conducting the sampling in the major health centers distributed around the greater Kigali metropolitan area.

### Variables

The outcome variable of interest in this study is developmental delay, which is defined as any single developmental domain (communication: child’s verbal and non verbal communication skills; gross motor: child’s ability to stand, walk and run; fine motor: child’s hands and finger movement; problem solving: child’s ability to play with toys and solve problems; and personal social skills: child’s self help skills and interactions with others) score falling below the ASQ-3 standardised lower cutoff points [25].

The ASQ-3 is a standardized screening tool used throughout the world: it consists of 30 age-specific questions in five domains that is completed by caregivers and resulting in a score reflecting the child’s status of being either on-track, needing monitoring due to potential concern (up to 1 standard deviation below the mean), or below cutoff, which is indicative of need for professional evaluation due to the high possibility of disability based on scores two or more standard deviations below the mean. In this study, children in the on-track and monitoring groups are considered normally developing while those below the cutoff are defined as having developmental delay [25].

**Explanatory variables** include Economic Categories I, II, and III which are Rwandan specific levels of household income and economic status (ubudehe category), where those in Level I earn less than those in Level III [29]. A caregiver is considered married if the child’s parent live with the partner, and single if the parent lives alone with the child. Children coming from Kigali city had their district of origin recorded, while children from outside Kigali have been classified in the “other” category.

The **continuous variables** of height, current weight, and head circumference have been measured on the day of DD screening by the data collectors and plotted on the standard WHO growth charts. The terms stunted or microcephalic refer to measurements with less than 2 standard deviations below the mean for age and sex [26]. We use the WHO definition of low birth weight (LBW) as a birth weight less than 2.5 kg, and preterm as a delivery at gestational age (GA) less than 37 weeks. The mother’s age in this study was the age of the mother on the day of the screening.

### Data collection process

Developmental data were collected using an official Kinyarwanda translation of the ASQ-3. The tool has gone through a validation process with the publisher to adapt the screening tool to Rwanda, and it has been purchased from Brookes Publishing Co., Inc. There are 21 age-specific ASQ-3 forms, and in this study, the 10 month form was used with 9–10 months old children and the 16 month form with those 15–16 months old. The questions were completed by parents upon entry to the health center and after consent was obtained, assisted by data collectors and the PI if a parent requested help, and were analysed immediately by the PI and entered into the database.

Anthropometric measures of weight, length, head circumference and middle upper arm circumference (MUAC) as well as the demographic and medical information were conducted by three trained data collectors and entered into the Google form database by the PI. Parents of children with suspected developmental delay or other medical comorbidities who might benefit from a pediatric visit were referred from the health center to pediatric consultants at the local district hospital.

### Data processing and analysis

Data collected on Google forms were migrated to EpiData V3.1 and then exported to SPSS 25 (IBM, New York, United States) for analysis. Descriptive analysis and categorical data are presented using frequency and percentages. Binary logistic regression was used to study the association between the outcome and possible risk factors or predictors. Factors with a p-value < 0.2 have been included in a multivariable logistic regression analysis to minimize confounding factors and to determine more accurate levels of association with developmental delay. Crude and AOR with a 95% confidence interval (CI) were computed. Multicollinearity and the Hosmer-Lemeshow test for model fitness have been performed after multivariable logistic regression and the Mean VIF (variance inflation factor) was 1.18 with a non-statistically significant p-value of 0.490 after the goodness-of-fit test. A p-value less than 0.05 was considered statistically significant.

### Data quality assurance

The principle investigator, a paediatric resident, provided training to the data collectors and monitored the data entry daily. The questionnaire and methodology were successfully pre-tested on 19 mother/infant pairs at the Nyiranuma Health Center, with no need to modify the process or further train the collectors. The ASQ-3 in Kinyarwanda is the copywritten, official and tested version purchased from Brookes [20].

## Results

**Fig. 1:**
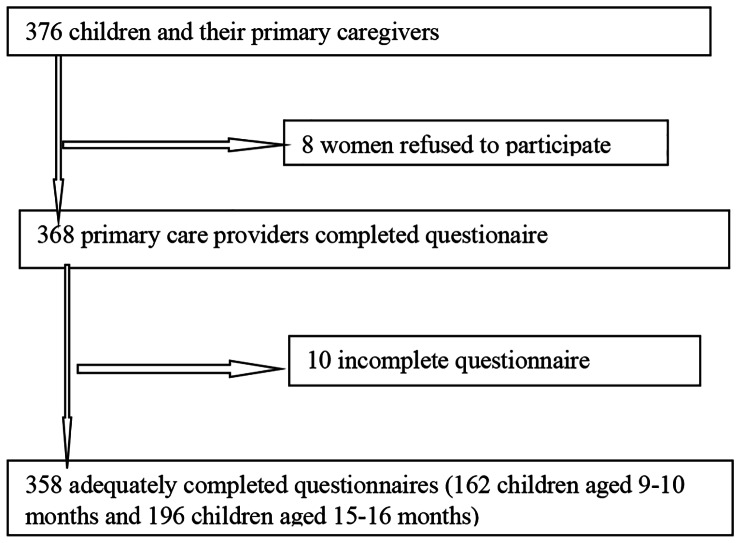
Flow chart of participantsselection

376 mothers/family care givers and their children were invited to participate in this study, and 358 adequately completed ASQ forms were analysed (Fig. 1).

A slight majority of children were male in both age groups, with 84 (51.5%) at 9–10 and 101 (51.5%) at 15–16 months. A majority of participants had a normal birthweight ≥ 2.5 kg with 146 (90.1%) at 9–10 and 185 (94.4%) at 15–16 months (Table 1).

Table 2 demonstrates that the majority of mothers were married 263 (73.5%), half of them were interviewed at the single Gasabo District center 188 (52.5%), almost all 321 (89.7%) had medical insurance, half were in the Rwandan middle class (economic category II) 190 (53.1%), a minority 37 (10.3%) had university degrees, very few were HIV positive 15 (4.2%) or smokers 3 (0.8%), and 37 (10%) used an undefined amount of alcohol at some time in pregnancy.

**Fig. 2:**
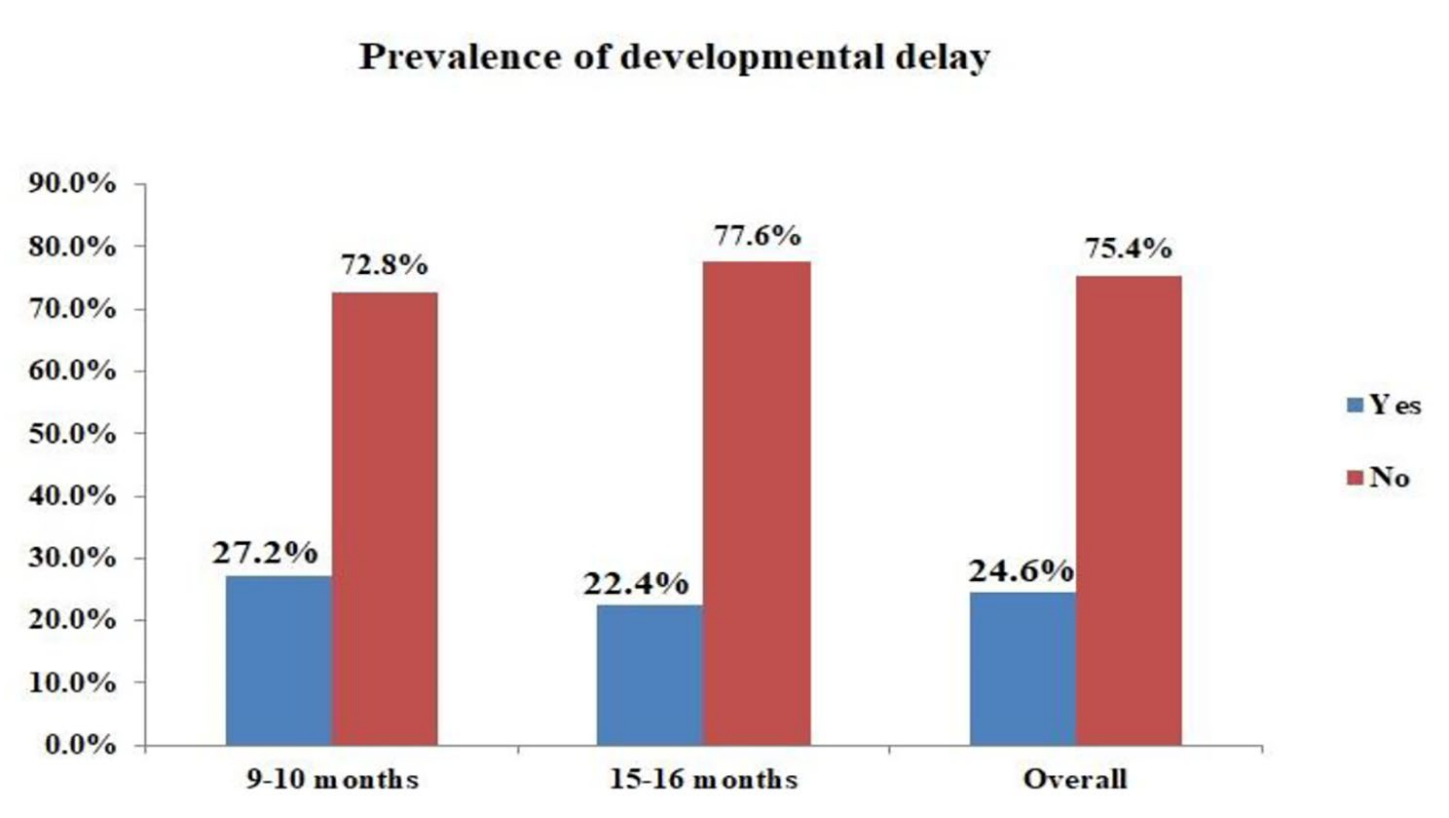
Age specific and overall prevalence of developmental delay

In the combined group of 358 children, the percentage of children with suspected DD in any one of the five domains varied between a low of 1.7% for personal social and 12.8% for gross motor. There existed a greater spread in those screening at-risk at 9 months (1.9–20.4%) compared to the older 15 month cohort (1.5–11.2%), reflecting the rapid improvement in gross motor skills (20–12.8%). The problem-solving domain was the most challenging for the older children at 11.2%, while communication scores were consistent between the groups (Table 3). Some children scored below the cut-off in multiple areas, and the overall prevalence of infants falling into the below-cutoff category in one or more domains was 27.2% at 9 months and 22.4% at 15 months with an overall prevalence of 24.6%, 95% CI (20.1, 29.1) (Fig. 2).

**Table 1:** Socio-demographic characteristics of enrolled children and their mothers (n = 358)

Characteristics	Children			
	9–10 months(n = 162)		15–16 months (n = 196)	
	n	%	N	%
**Gender**				
Female	78	48.1	95	48.5
Male	84	51.9	101	51.5
**Birth weight**				
< 2.5 kg	16	9.9	11	5.6
≥ 2.5 kg	146	90.1	185	94.4
**Height**				
Normal	159	98.1	195	99.5
Stunted	3	1.9	1	0.5
**Head circumference**				
Normocephalic	158	97.5	192	98.0
Microcephalic	4	2.5	4	2.0
**Current weight**				
Normal	157	96.9	194	99.0
Wasted	5	3.1	2	1.0
**Gestational age at delively**				
Term	152	93.8	192	98.0
Preterm	10	6.2	4	2.0
**Mother’s age**				
≤ 35 years	149	92.0	171	87.2
> 35 years	13	8.0	25	12.8
**Number of children in the household**				
1–3 children	137	84.6	167	85.2
≥ 4 children	25	15.4	29	14.8
**Started breastfeeding on first day of life**				
Yes	152	93.8	187	95.4
No	10	6.2	9	4.6
**Admitted to hospital in first month of life**				
No	148	91.4	182	92.9
Yes	14	8.6	14	7.1

**Table 2:** Socio-demographic characteristics of the caretakers of recruited children (n = 358)

Characteristics	n	%
**Marital status**		
Married	263	73.5
Single	94	26.3
Widower	1	0.3
**Relationship with the child**		
Mother	338	94.4
Father	7	2
Other	13	3.6
**District of origin**		
Nyarugenge	92	25.7
Gasabo	188	52.5
Kicukiro	71	19.8
Out of Kigali	7	2
**Economic category**		
Category I	21	5.9
Category II	190	53.1
Category III	147	41.1
**Medical insurance**		
Yes	321	89.7
No	37	10.3
**Education background**		
Primary	158	44.1
Secondary	141	39.4
University	37	10.3
None	22	6.1
**Disease during pregnancy**		
None	342	95.5
HIV	15	4.2
Syphilis	1	0.3
**Alcohol and tobacco consumption during pregnancy**		
None	318	88.8
Alcohol	37	10.3
Tobacco	3	0.8

**Table 3:** Domain specific developmental status of study subjects using the 10 and 16 month ASQ-3 forms

Domain	Children					
	9 − 1 0months		15–16 months		Overall total	
	n (162)	%	n (196)	%	n (358)	%
**Communication**						
Below cutoff	4	2.5	5	2.6	9	2.5
Close to cutoff	13	8	26	13.3	39	10.9
Above cutoff	145	89.5	165	84.2	310	86.6
**Gross motor**						
Below cutoff	33	20.4	13	6.6	46	12.8
Close to cutoff	20	12.3	19	9.7	39	10.9
Above cutoff	109	67.3	164	83.7	273	76.3
**Fine motor**						
Below cutoff	10	6.2	20	10.2	30	8.4
Close to cutoff	49	30.2	60	30.6	109	30.4
Above cutoff	103	63.6	116	59.2	219	61.2
**Problem solving**						
Below cutoff	5	3.1	22	11.2	27	7.5
Close to cutoff	45	27.8	46	23.5	91	25.4
Above cutoff	112	69.1	128	65.3	240	67
**Personal-social**						
Below cutoff	3	1.9	3	1.5	6	1.7
Close to cutoff	21	13	9	4.6	30	8.4
Above cutoff	138	85.2	184	93.9	322	89.9

Note: *below cutoff refers to children whose scores at more than 2 standard deviations below the mean indicate a need for further assessment because they have suspected developmental delay. Close to cutoff refers to children who need ongoing monitoring with scores between 1 and 2 standard deviations below the mean. Above cutoff indicates typical development*

Bivariate analysis demonstrates a strong association between suspected DD and three variables. Prior preterm infants were more likely to screen at high risk of having DD compared to term infants (OR = 8.52; 95% CI = 2.60–27.90); prior low birth weight infants of < 2.5 kg were at higher risk for DD compared with normal birth weight children (OR = 2.68; 95% CI = 1.20–5.99); and children from Nyarugenge district had an increased risk of developmental delay compared to children from Gasabo district (OR = 2.19; 95% CI = 1.24–3.90). Interestingly, there was no demonstrated association between prenatal alcohol exposure and delay (Table 4).

**Table 4:** Bivariate analysis of association between DD and potential predictors

Predictor	DD		OR (95% CI)	P value
	Yes	No		
**Birth weight**				
< 2.5 kg	12 (44.4%)	15(55.6%)	2.68 (1.20–5.99)	**0.** ***016***
≥ 2.5 kg	76 (23.0%)	255 (77.0%)	**Ref**	
**Current weight**				
Normal	84 (23.9%)	267 (76.1%)	**Ref**	
Wasted	4 (57.1%)	3 (42.9%)	4.2 (0.93–19.3)	0.*062*
**Household size**				
1–3 children	75 (24.7%)	229 (75.3%)	1.03 (0.52–2.03)	0.925
4 and more children	13 (24.1%)	41 (75.9%)	**Ref**	
**Mother’s education**				
Primary/None	43 (23.9%)	137 (76.1%)	**Ref**	
Secondary/University	45 (25.3%)	133 (74.7%)	1.07 (0.66–1.74)	0.76
**Mother’s age**				
≤ 35 years	76 (23.8%)	244 (76.3%)	**Ref**	
> 35 years	12 (31.6%)	26 (68.4%)	1.48 (0.71–3.07)	0.292
**Marital status of the mother**				
Married	60 (22.8%)	203 (77.2%)	**Ref**	
Single	28 (29.5%)	67 (70.5%)	1.41 (0.83–2.39)	0.197
**Economic category**				0.672
Category I	6 (28.6)	15 (71.4)	1.10 (0.0.40–3.1)	
Category II	43 (22.6)	147 (77.4)	0.8 (0.50–1.33)	
Category III	39 (26.5)	108 (73.5)	**Ref**	
**District of origin**				**0.022**
Nyaraugenge	30 (32.6)	62 (67.4)	2.19 (1.24–3.90)	
Gasabo	34 (18.1)	154 (81.9)	**Ref**	
Kicukiro	21 (29.6)	50 (70.4)	0.87 (0.44–1.67)	
Out of Kigali	3 (42.9)	4 (57.1)	1.55 (0.32-7.40-)	
**Medical insurance**				0.715
Yes	78 (24.3)	243 (75.7)	**Ref**	
No	10 (27.0)	27 (73.0)	1.2 (0.53–2.49)	
**Alcohol/smoking**				
No	75 (23.6%)	243 (76.4%)	**Ref**	
Yes	13 (32.5%)	27 (67.5%)	1.56 (0.76–3.17)	0.22
**Gender**				
Female	46 (26.6%)	127 (73.4%)	1.23 (0.76–1.99)	0.394
Male	42 (22.7%)	143 (77.3%)	**Ref**	
**Gestational age at delivery**				
Term	78 (22.7%)	266 (77.3%)	**Ref**	
Preterm	10 (71.4%)	4 (28.6%)	8.52 (2.6–27.9)	**< 0.** ***001***
**Breastfeeding on the first day**				
Yes	81 (23.9%)	258 (76.1%)	**Ref**	
No	7 (36.8%)	12 (63.2%)	1.85 (0.71–4.87)	0.208
**Admitted in hospital in the first month of life**				
No	79 (23.9%)	251 (76.1%)	**Ref**	
Yes	19 (32.1%)	19 (67.9%)	1.50 (0.65–3.46)	0.336

Multivariable analysis (Table 5) of the variables that showed p < 0.2 significance in the binary logistic regression (birth weight, gestational age at delivery, current weight, marital status of the mother and district of origin) resulted in gestational age and district of origin being factors associated with DD. Consistent with the literature, preterm children were at higher risk of being screened below the cutoff compared to term children (AOR = 8.60; 95% CI = 2.70–28.50). Children from Nyarugenge District have an increased risk of DD compared to children from Gasabo district (OR: 2.15; 95% CI = 1.20–3.87).

**Table 5:** Multivariable analysis of the predictors of DD using variables of significance < 0.2 from the bivariate logistic regression analysis

Risk factors	N	Adjusted OR	95%CI	P-value
**Gestational Age at delivery**				**< 0.001**
Term Preterm	344 (96.1) 14 (3.9%)	Ref 8.6	2.7–28.5	
**Birth weight**				0.984
< 2.5 kg ≥ 2.5 kg	27 (7.5%) 331 (92.5%)	1.01 **Ref**	0.34–3.01	
**Current weight**				0.223
Normal Wasted	351 (98%) 7 (2.0%)	**Ref** 2.83	0.54–14.91	
**District of orgin**				**0.046**
Nyarugenge Gasabo Kicukiro Out of Kigali	92 (25.7%) 188 (52.5%) 71 (19.8%) 7 (2%)	2.15 **Ref** 0.89 1.75	1.20–3.87 0.44–1.76 0.37–8.40	
**Marital status**				0.698
Married Single	263 (73.5%) 95 (26.5%)	**Ref** 1.12	0.63–1.98	

## Discussion

This study successfully screened young children from three districts of the capital city of Rwanda and our data reveal that 24.6% (95% CI = 20.1–29.1) of children between 9 and 16 months of age screened positive for DD in at least one of the five critical areas of development examined. This 24.6% prevalence is lower than the 2019 Rwandan demographic health survey prevalence of 33% among urban Rwandan children aged 3–5 years old using a different tool and geographic settings [27]. It is significantly lower than the 67.4% prevalence using the same ASQ-3 tool with children at high risk of DD between 1 and 3 years old who were ex-premature babies in a rural Rwandan Neonatology Intensive Care Unit follow-up clinic [20]. There are few comparable studies in Sub-Saharan Africa: this Rwandan prevalence is lower than the 44.6% in rural Ghanaian children under 5 years of age, and higher than the 11.7% in a rural Malawian preschool setting using different screening tools [28–30].

Similar studies have been conducted on other continents and our 24.6% is far lower than the 51% found among 4–24-month Indian children, where 40% of them were identified as high risk [31]. A comparable prevalence of 28.8% was found among 9–30 month Chilean children using a similar screening tool [23].

The subcategories of delay, or domains, are also important to consider. In our combined cohort, the prevalence of delay in different domains ranged between1.7% and 12.8%. A 2014 UNICEF baseline evaluation that studied rural Rwandan children as part of an early child development and family service (ECD + F) study demonstrated delays of 10–35% in a younger 0–11 month cohort and 13–33% in an older 24–45 month cohort. This higher prevalence and range compared to our urban study participants may reflect the family conflict, poverty, and higher proportion of illiterate primary care providers that was noted in their study population [21].

There is a striking similarity between our urban cohort and the rural UNICEF ECD + F group regarding rapid improvement of gross motor skills as children age: 20.4% of our younger cohort fell below the gross motor cutoff and that dropped to 6.6% in the older group. A similar large improvement in motor skills was seen in the rural ECD + F children and may reflect the fact that younger Rwandan children are carried alomost constantly up to 9 or 10 months of age and then strongly encouraged to stand and walk.

Problem solving (11.2%) was the domain that challenged the majority of our older age group. Interestingly, communication scores were very similar at both ages and are similar to a South African study in which young infants scored equally well as children in western countries. The South African authors suggested that it is due to the day-to-day practices of African mothers who carry their babies on the mother’s body, feeding on demand, immediately pacifying babies when they cry, co-sleeping, and the exposure of babies to a wide social network of kin and family [32]. Likewise, similar findings were reported in an Iranian study where their children scored well compared to children in HIC in the areas of communication and problem solving during the first 3 years of life [33].

Two factors emerge in this study as prominent predictors of falling below the ASQ-3 cutoff and indicative of delay. The first is prematurity and is consistent with findings in Rwanda, Chile, Canada, and Norway and is proposed to be due to stresses on the immature brain, perinatal risk factors, and high susceptibility to environmental exposures [34, 20, 35–37]. These findings lend urgency to the importance of implementing screening and intervention programs while concurrently improving neonatal services in Rwanda.

The second predictive factor is place of residence, with our study highlighting the difference in levels of developmental delay between children from different parts of the city. Further study is needed to elucidate which factors contribute to the children in Nyarugenge screening for delay at twice the rate of those in Gasabo: The Rwandan demographic and health survey hints at greater numbers of less educated adults, more infant stunting, higher teenage pregnancy, and less insurance coverage in Nyarugenge compared to other neighbouring districts [38]. In contrast to the strong association with prematurity, birth weight did not appear to be associated with delay after multivariate analysis, which surprised us since a systematic review of African children had earlier made that association [39].

### Limitation of the study

Rwanda is a largely rural country and these results, generated from studying urban children, many of whom come from families in the middle economic level, may not be generalizable to the broader population. Caregiver recall of gestational age at delivery and birth weight is not consistent as evidenced by some mothers referring to the vaccination card in order to remind themselves of the birth weight. We do not feel that a data collector helping a caregiver to read and understand questions is a limitation since that occurred in the development of the ASQ-3 and is employed as a routine in many clinical settings when there are literacy barriers. Assigning the label of developmental delay based on a single screening is not something that the ASQ-3 user’s guide endorses but for the purposes of this study it provides us a rough estimate and not a clinically accurate diagnosis.

#### Study strength

Regardless of the limitations, this study contributes valuable data and methodology for health planners in Rwanda and neighbouring East African nations by utilising an internationally validated tool that has been used around the world.

## Conclusion

This study demonstrates a strong association between prematurity and poorer districts in the Kigali metropolitan area with developmental delay in 9–16-month urban Rwandan children. Improvements in neonatal care throughout Rwanda and other LMICs must be paired with improved developmental surveillance and interventions for the greater number of higher risk children surviving. Further work will be needed to identify which factors in Kigali’s poorer districts lead to increased rates of delay so that scarce resources are prioritised where they are most needed. This study demonstrates that the need exists and that it will take effort to devise even targeted intervention programs that are culturally appropriate and economically feasible and respond to the aforementioned call to action by the World Bank.

## Data Availability

The datasets used and/or analyzed during the current study are available from the corresponding author on reasonable request.

## Abbreviations

ASQ-3: Ages and Stages Questionnaire
CMHS IRB: College of Medicine and Health Sciences Institutional Review Board
RDHS: Rwanda Demographic Health Survay
DD: Developmental Delay
UNICEF: United Nations Children’s Fund
